# The Effects of Concurrent Resistance and Endurance Training Follow a Specific Detraining Cycle in Young School Girls

**DOI:** 10.2478/v10078-011-0064-3

**Published:** 2011-10-04

**Authors:** Albano Santos, Daniel A. Marinho, Aldo M. Costa, Mikel Izquierdo, Mário C. Marques

**Affiliations:** 1 Department of Sport Sciences, University of Beira Interior (UBI), Covilhã, Portugal; 2 Research Centre for Sport Sciences, Health and Human Development, Vila Real, Portugal; 3 Department of Health Sciences, Public University of Navarre, Navarre, Spain

**Keywords:** Youth, Strength, Endurance, School, Experimental, weight training, detraining

## Abstract

The purpose of this study was to compare the effects of an 8-week training period of strength training alone (GR), or combined strength and endurance training (GCOM), followed by 12-weeks of de-training (DT) on body composition, power strength and VO_2_max adaptations in a schooled group of adolescent girls. Methods: Sixty-seven healthy girls recruited from a Portuguese public high school (age: 13.5+1.03 years, from 7^th^ and 9th grade) were divided into three experimental groups to train twice a week for 8 wks: GR (n=21), GCOM (n=25) and a control group (GC: n=21; no training program). Anthropometric parameters variables as well as performance variables (strength and aerobic fitness) were assessed. Results: No significant training-induced differences were observed in 1kg and 3kg medicine ball throw gains (2.7 to 10.8%) between GR and GCOM groups, whereas no significant changes were observed after a DT period in any of the experimental groups. Significant training-induced gains in CMVJ (8 to 12%) and CMSLJ (0.8 to 5.4%) were observed in the experimental groups. Time of 20m significantly decreased (GR: −11.5% and GCOM: −10%) after both treatment periods, whereas only the GR group kept the running speed after a DT period of 12 weeks. After training VO_2_max increased only slightly for GCOM (4.0%). No significant changes were observed after the DT period in all groups, except to GCOM in CMVJ and CMSLJ. Conclusion: Performing simultaneous strength and endurance training in the same workout does not appear to negatively influence power strength and aerobic fitness development in adolescent girls. Indeed, concurrent strength and endurance training seems to be an effective, well-rounded exercise program that can be prescribed as a means to improve initial or general strength in healthy school girls. De-training period was not sufficient to reduce the overall training effects.

## Introduction

Strength training is defined as a specialized method of conditioning that involves the progressive use of a wide range of resistive loads and a variety of training modes (e.g., free weights, weight machines, elastic cords, medicine balls, and body weight) designed to enhance health, fitness and sports performance (0). Scientific evidence indicates that strength training should be part of a comprehensive health maintenance (Faigenbaum, 2007) and physical performance (Anderson and Haraldsdottir, 1995) effective strategy for youth, as long as it is carefully prescribed and monitored (Simons-Morton et al., 1993; Sharma, 2006; Izquierdo et al., 2010). Further, female participation in sport has increased dramatically over the previous 20 years in a variety of events. However, despite the increase in female physical activity (PA) regular programs, there is a paucity of research on performance characteristics of female adolescents and to the authors’ knowledge few data are available for young school girls (Sweeting, 2007; Faigenbaum et al., 2009).

School girls have been described as less active than their male age-peers (Nielsen and Andersen, 2003; Faigenbaum, 2007) and become even less physically active as they are going through adolescence (Twisk et al., 2000; Sweeting, 2007). Nevertheless, it was reported by several studies that physical activity levels of children aged 13 to 15 years old are positively related with physical fitness (Malina, 2001). Moreover, there is strong evidence that school-based interventions are effective to promote PA levels (Faigenbaum et al., 1996; Strong et al., 2005; Sweeting, 2007) and, therefore, school seems to provide an excellent setting to enhance its levels by implementing physical fitness programs.

Both strength and endurance training are often performed concurrently in most exercise programs in wellness, fitness and rehabilitative settings, in an attempt to reach different physical fitness goals (Anderson and Haraldsdottir, 1995). Several studies using young adult sample, have shown that simultaneously performing strength and cardiovascular training, the strength gains achieved by strength training alone may be impaired (Kraemer et al., 1995). Unfortunately, few authors have examined the effects of concurrent strength and endurance training on different days (Sale et al., 1990), on the same day (Abernethy and Quigley, 1993; Volpe et al., 1993) or a compound of both methods (Hunter et al., 1987). Researches in a school environment, concerning this issue, are even scarcer (Izquierdo et al., 2010). Moreover, to our best knowledge, no study prior to ours had studied the effects of power training with concurrent power and endurance training on muscular strength development in a large sample of non-athlete adolescent girls.

Physical activity interruption because of illness, injury, holidays, or post-season break occurring through life or other factors are normal situations in any kind of sport (0; Garrido et al., 20100). The magnitude of this reduction may depend upon the length of the detraining period in addition to training levels attained by the subject (0). However, the detraining period and its consequences are not well reported in sports literature during puberty. Additionally, a period of strength training cessation can also produce a positive delay transformation to enhanced sports specific performance (0). In fact, it has been shown that physical fitness improves during the school year (yr), with little or no changes during the summer holidays (0). Another study (0) could observed that girls can significantly reduce cardiorespiratory fitness after the holiday period. However, the detraining period and its consequences are not well reported in the scientific community, or within a group of school girls (0; 0). Furthermore, the effects of training may not manifest soon after the training but may appear later.

According to the above mentioned, we hypothesized that concurrent strength and endurance training would have a bigger positive effect on muscular strength development of untrained school girls compared with the results found when strength was trained alone. We also hypothesized that both strength training and concurrent strength and endurance training groups would keep some strength gains after a training break. Therefore, the main purpose of the current study was twofold: (i) to analyze the effects of strength training alone, or combined strength and endurance training on body composition, strength and cardiovascular markers on a sample of healthy schoolgirls and, (ii) to assess the effects of a de-training period on strength and endurance performance.

## Methods

### Experimental design

Sixty-seven healthy girls (13.5±1.03 years old) recruited from a Portuguese public high school were divided into two experimental groups (to train 2 times per week for 8 weeks) and one control group as follows: one group performing strength training only (GR: n=21); another group performing combined strength and endurance training (GCOM: n=25); an additional group as control (GC: n=21; without training program). All subjects attended physical education classes twice a week, with a duration of 45 min and 90 min each class respectively. In these classes, students took part in various sports (gymnastics drills, soccer, basketball and volleyball) with a clear pedagogical focus. As such, according to other researchers (Simons-Morton et al., 1993; Silva et al., 2010) the physical activity intensity is considered low to moderate. Participants in all groups were asked to maintain normal eating and physical activity patterns over the duration of the study. This procedure was the same as Lubans et al. (2010).

Beyond to physical education classes, and after a 10 min warm up period (7 min running with an intensity sufficient to raise breath rate, 3 min stretching and joint specific warm up), both experimental groups were submitted to a strength training program composed by: 1 and 3 kg medicine ball throws; jumps onto a box (from 0.4 m to 0.6 m); plyometric jumps above 0.4–0.6 m of height hurdle and; sets of 30 to 40m speed running. The GCOM group was further subjected to a 20m shuttle run exercise (0).

This endurance task, which occurred immediately after the strength training session, was developed based on an individual training volume - set to about 75% of the established maximum aerobic volume achieved on a previous test.

After 4 weeks of training, GCOM subjects were reassessed using 20m shuttle run tests in order to readjust the volume and intensity of the 20m shuttle run exercise. All participants were familiarised with power training tests (sprints, jumps and ball throws) as well as with the 20m shuttle run test. A more detailed analysis of the program can be found in table 1.

All sample groups were assessed for upper and lower body power strength (overhead medicine ball throwing and counter movement vertical jump, respectively), running speed (20 m sprint run) and VO_2_max estimate (20 meters shuttle run test) before and after 8-weeks of training.

In order to evaluate the DT effects, all individuals were reassessed 12 weeks after training has ceased. The DT period was coincidental with summer holidays. Throughout this period, the subjects reported their non-involvement in regular exercise programs for developing or maintaining strength and endurance performance. The testing assessment procedures were always conducted in the same indoor facility, at the same hour and on the same weekday (from March to September of 2010). Data collection was performed by the same investigator and after a general warm-up of 10 minutes.

### Subjects

A sample of 67 healthy girls recruited from a Portuguese public high school (from 7^th^ and 9^th^ grades) was used in this study. To fulfill the ethical procedures of the Helsinki statement (WMA Declaration of Helsinki, 2008), a consent form was obtained prior to all the testing from parents or a legal guardian of the adolescents. Efforts were made to pick subjects for making comparable groups. Maturity level based on Tanner stages (Duke et al., 1980) was self-assessed. Students were asked to answer to an image with corresponding legend questionnaire. Students answered the questionnaire in an individual booth without interference from their teachers or school friends. There were no significant differences (p>0.05) between groups for age or Tanner stages, neither in strength or endurance fitness performances at the beginning of the protocol. No subject had regularly participated in any form of strength training program prior to this experiment. The following exclusion criteria were used: subjects with a chronic paediatric disease or with an orthopaedic limitation.

### Anthropometrical Variables

Total height (m) was assessed according to international standards for anthropometric assessment (0), with a Seca 264 Stadiometer (Hamburg, Deutschland). Weight and body fat were assessed using a Tanita body composition analyser; model TBF-300 (Tanita Corporation of America, Inc, Arlington Heights, IL) with a range of ratio 1%–75%. These two parameters were assessed prior to any physical performance test. Subjects were measured wearing shorts and t-shirts (shoes and socks were asked to be removed).

### Overhead Medicine Ball Throwing

An overhead medicine ball throw was used to evaluate the upper body ability to generate muscular actions at a high rate of speed. Prior to baseline tests, each subject underwent one familiarization session and was counselled on proper overhead throwing with different weighted balls. Pre-tests, post-tests and de-training measurements were taken on maximal throwing velocity using medicine balls weighing 1kg (perimeter 0.72m) and 3kg (perimeter 0.78m). A general warm-up period of 10 minutes, which included throwing the different weighted balls, was allowed. While standing, subjects held medicine balls with 1 and 3kg in both hands in front of the body with arms relaxed. The students were instructed to throw the ball over their heads as far as possible. A counter movement was allowed during the action. Five trials were performed with a one-minute rest between each trial. Only the best throw was used for analysis. The ball throwing distance (BTd) was recorded to the closest cm as proposed by van Den Tillaar & Marques (2009). This was possible as polyvinyl chloride medicine balls were used and when they fall on the Copolymer Polypropylene floor they make a visible mark. The ICC of data for 1kg and 3 kg medicine ball throwing was 0.94 and 0.93, respectively.

### Counter Movement Vertical Jump (CMVJ)

The standing vertical jump is a popular test of leg power and is routinely used to monitor the effectiveness of an athlete's conditioning program. The students were asked to perform a counter movement jump (with hands on pelvic girth) for maximum height. The jumper starts from an upright standing position, making a preliminary downward movement by flexing at the knees and hips; then immediately extends the knees and hips again to jump vertically up off the ground. Such movement makes use of the stretch-shorten cycle, where the muscles are pre-stretched before shortening in the desired direction (0). It was considered only the best performance from the three jump attempts allowed. The counter movement vertical jump has shown an ICC of 0.89.

### Counter Movement Standing Long Jump (CMSLJ)

Each participant completed three trials with a 1-min recovery between trials using a standardised jumping protocol to reduce inter-individual variability. From a standing position, with the feet shoulder-width apart and the hands placed on the pelvic girth, the girls produced a counter movement with the legs before jumping horizontally as far as possible. The greatest distance (meters) of the two jumps was taken as the test score, measured from the heel of the rear foot. A fiber-glass tape measure (Vinex, MST-50M, Meerut, India) was extended across the floor and used to measure the horizontal distance. The counter movement standing long jump has shown an ICC of 0.96.

### Sprint Running

The time to run 20m was obtained using a Brower Timing System (Utah, USA). At the start each subject trod the cell pad. The time to run the distance was recorded using a digital and automatic chronometer commanded by the cell pad and a pair of photocells positioned above the 20m line. All subjects were encouraged to run as fast as possible and to decelerate only after listening to the beep emitted by the last photocells pair. Each student repeated the same procedure for 3 attempts and only the best time reached was recorded. The sprint running (time) has shown an ICC of 0.85.

### 20 Meter Shuttle Run (VO_2max_)

This test involves continuous running between two lines (20m apart in time) to recorded beeps. The time between recorded beeps decreases each minute (level). We chose to use the common version that has an initial running velocity of 8.5 km/h, which increases by 0.5 km/h each minute (0). The final students score was based on the level and number of shuttles reached before they were unable to keep up with the audio recording. Estimated VO_2max_ (ml.kg^−1^.min^−1^) was calculated by the Léger's equation (0). The 20m Shuttle Run test has shown an ICC of 0.91.

### Statistical analyses

Standard statistical methods were used for the calculation of the means and standard deviations (SD). One-way analysis of variance (ANOVA) was used to determine any differences among the three groups’ initial strength, endurance, running speed and anthropometry. The training related effects were assessed using a two-way ANOVA with repeated measures (groups x moment). Selected absolute changes were analyzed via one-way ANOVA. The p ≤ 0.05 criterion was used for establishing statistical significance.

## Results

At baseline, no significant differences were observed between groups for any of the pre-training anthropometrics and performance variables (p>0.05). Body fat (BF) significantly decreased (p<0.01) from the pre-training to the post-training period in all groups (Table 2). No significant changes were observed for height, body weight and body mass index (BMI) in any of the groups. Only GCOM increased significantly 1kg and 3kg ball throw distance (p<0.05). GR increased significantly 3kg ball throw distance (p<0.05) (Table 3). The CMVJ height remained stable after the training program for group GR (0%; ns) whereas GR (+8%; 0.01) and GCOM (+12%; 0.00) significantly increased CMVJ height after the training program. Both experimental groups also increased their performance in CMSLJ after the training program: GR (+0.8%; 0.04) and GCOM (+5.4%; 0.01). GC (−2.3%; ns) didn’t change significantly CMSLJ height in the same period. The time to run 20m significantly decreased in GR (−11.5%, p=0.00) and GCOM (−10%, p=0.00), whereas remained constant in GC. The amount of changes was similar in both GR and GCOM groups. Finally, the VO_2_max increased significantly in both GC (+3.2%, p<0.05) and GCOM (+4.0%, p<0.01), whereas it remained unchanged in GR group.

The detraining period resulted in an increase in body weight (+1.6%, p<0.04) for GCOM (Table 3), whereas remained constant for the GR and GC groups. Body height increased significantly for GR (+0.2%, p<0.03).

This endurance task, which occurred immediately after the strength training session, was developed based on an individual training volume - set to about 75% of the established maximum aerobic volume achieved on a previous test.

No significant changes were observed in the 1kg and 3kg medicine ball throw gains after the DT period in any of the experimental groups (Table 3). Additionally, table 3 shows that all groups had significantly lower scores in the vertical jump height after DT period: less 23.1% for GC (p=0.00), less 3.7% for GR (p=0.02) and less 14.3% for GCOM (p=0.00). Significant differences were found between GC and GR as well as between GR and GCOM groups. Both GC (+1.6%; ns) and GR (−3.8%; ns) didn’t change their CMSLJ performance after the de-training period. However, GCOM (−4.4%; 0.00) has reduced CMSLJ height in the same period. The time to run 20m decreased in GC and GCOM (1.2% and 1.9%, respectively), yet no significant differences between groups were observed after DT. Estimated VO_2_max remained unchanged after DT period in all groups.

## Discussion

### Training period

The primary findings of the study showed that concurrent strength and cardiovascular training may be a positive training stimulus to induce power strength and aerobic fitness development and also showed an extremely positive effect on body fat loss in adolescent school girls. Therefore, the present results may suggest that concurrent strength and endurance training seems to be an effective, well-rounded exercise program that can be used as a means to improve initial or general strength in healthy school girls.

In GCOM, the magnitude of decrease observed in BF was significantly greater (−11.4%, p=0.01) than that observed in GR (−6.2%; p=0.03). However, we did not find any change in body weight and BMI for any group. These results suggest a major positive effect of concurrent strength and endurance training over body fat loss occurs. This could be related to the fact that aerobic exercise can contribute an increase on fat metabolism. In fact, it is known that insulin sensitivity increases with aerobic training and also has an effect on glucose transportation; insulin has an anabolic effect on fat storage in the fat cells (Nielsen and Andersen, 2003).

Insulin affects appetite regulation through the change in substrates in the blood. Insulin sensitivity may therefore be one of the key mechanisms behind the association found between body composition and fitness (Nielsen and Andersen, 2003). Furthermore, although the design of the training intervention of this study is different from research conducted by Watts et al. (2004), the current results are in agreement with their study results. Watts et al. (2004) examined 19 obese adolescents aged 12–16 years independent influence of 8 weeks of combined strength and aerobic training. Here, although bodyweight and BMI has not changed with exercise, significant improvements in central adiposity were observed following the 8-week circuit-training programme.

Moreover, the total body fat decreased but the majority of fat tissue mass was lost from the abdominal and trunk areas. Interestingly, subcutaneous (skinfold) fat measures did not change, even in these areas, suggesting that exercise training may beneficially modify body composition, with initial decreases in fat predominantly occurring from the viscera.

Upper power strength (e.g. the medicine ball throw with 1kg and 3kg), has significantly increased in both GCOM and GR group. This data may suggest a positive influence of strength training on power strength performance results, no matter with or without concurrent strength and endurance training. Concordantly to the upper body strength results, the power of lower limbs revealed by the CMVJ and CMSLJ performance has changed for both experimental groups. To our best knowledge, very few studies have compared the effects of different methods of organising training workouts. For example, Sale et al. (1990) observed that concurrent strength and endurance training applied on separate days produced gains superior to those produced by concurrent training on the same day. Although the training programs were held otherwise constant, alternate-day training was more effective in producing maximal leg press strength gains than same-day training. This suggests that the interference effect may also be true when the overall frequency and/or volume of training are higher than in this particular study. Briefly, the results do not demonstrate the universality of the interference effect in strength development when strength training is performed concurrently with endurance training in school girls. It is difficult to compare the results in scientific literature when studies differ markedly in their design factors including mode, frequency, intensity, volume of training, and training history of subjects (Izquierdo et al., 2010). Therefore, further research is required to investigate these causes and identify other possible mechanisms responsible for the observed inhibition in strength development after concurrent training (Watts et al., 2004).

Running speed increased significantly in all experimental groups. These results seem to indicate that additional endurance training does not have an additional effect over strength training to enhance running speed in young girls. On the other hand, all students approached various sports during Physical Education classes. Although physical activity intensity can be considered low to moderate, some sports (for instance, soccer and basketball) elicit high intensity performances (sprints) and low-intensity periods, which could have enhanced running speed performance.

Many people rationalise that concurrent training will give them the benefits of both strength and endurance training (Abernethy and Quigley, 1993). The fact that an inhibition in strength or endurance adaptation as a consequence of concurrent training has been reported (Volpe et al., 1993). The present study, however, could observe a significant enhancement in VO_2_max (ml.kg^−1^.min^−1^) for both GC and GCOM, suggesting that the endurance training program component was effective to a rising in aerobic fitness independently of the treatment group. Our data suggest that dependent variable selection can influence conclusions made with respect to changes in strength and endurance as a result of concurrent training. However, differences in the design of concurrent training interventions, such as mode, duration, and intensity of training, may influence whether any interference in strength or endurance development is observed. Clearly, the interaction between strength and endurance training is a complex issue, and it may still be possible to design specific concurrent training regimens that can minimize or possibly avoid any interference effects.

### Detraining period

To our best knowledge, no other study has established the effect of an 8-week school based endurance and strength training program and de-training on dynamic muscular strength and body composition in adolescent girls, performed additionally to the physical education lessons. Thus, it is difficult to compare the present relsults with other studies that have investigated physical training cessation because they differ markedly in a number of factors, including the sample and the method of measurement.

The detraining period was coincided with the summer holidays: 12 consecutive weeks. Thus, sample subjects had no formal physical activity (Physical Education lessons or institutional training programs) during this period. Despite that physical activity had decreased in an overall view, all groups kept body composition. Only the GCOM increased significantly in body weight (+1.6%) but not BF. Additionally, the biggest BF percentage loss was noticed in GCOM during the intervention period. Therefore, we can assume that the sustainment of BF obtained within the training programs participation is visible for several weeks after the programme has finished. Regarding to CMVJ, all groups had shown a significant loss of performance trend (p<0.02). However, in CMSLJ only GCOM had significantly reduced (p=0.00) performance during the de-training recess. This decrement is not surprising since GCOM had a higher increase (however not significantly different from GR) during the training period. In speed running a significant loss of performance was found in GC and GCOM, but not in GR. This loss was expected as speed running is strongly affected by the nervous system adaptation and phosphocreatine reserves. In the 1 and 3kg medicine ball throw distance test, no significant changes were observed for the experimental groups, despite an overall increase in performance, which means a more sustained effect of training in this power task. The control group had the worst performance marks for both the 1 and 3kg medicine ball throw distance test. Yet, only the 3kg medicine ball throw distance test, change was significant. For both variables, differences were found between GC and GR as well as between GC and GCOM. Thus, power strength gains from both training programs were kept after a DT period of 12 weeks, as strength is determined, among other factors, by muscular mass. Faigenbaum et al. (1996) results show that the 8 weeks of de-training led to significant losses of leg extension (−28.1 %) and chest press (−19.3%) strength whereas the control group strength scores remained relatively similar. Finally, the VO_2_max (ml.kg^−1^.min^−1^) remained stable for all groups, except for GCOM where a significant loss (−4.3%) was observed. Mujika and Padilla (2001) found that changes are more controlled in recently trained subjects (compared with highly trained subjects) in the short-term, but recently acquired VO_2_max gains are completely lost after training ceases for longer than 4 wks. Conversely, our results show that GCOM kept VO_2_max gains even after 12 wks of DT.

Overall, our results suggest that concurrent strength and endurance school-based training programs seem more effective on both strength and endurance fitness feature of age-school girls. In other words, our study indicates that concurrent training is an effective, well-rounded exercise program that can be set up as a means to improve initial or general strength in healthy school girls. Moreover, performing simultaneously strength and endurance training in the same workout does not impair strength development in young girls, which has important practical relevance for the construction of strength training in school-based programs. The de-training period was not sufficient to reduce the overall training effects. Future studies should examine the interference effects arising from the arrangement of strength and endurance training exercises (e.g., endurance training before strength training or vice versa) on strength.

## Figures and Tables

**Table 1:** Design of the training program performed

Exercises	Session 1	Session 2	Session 3	Session 4	Session 5	Session 6

Chest 1 kg Medicine Ball Throw ^1,2^	2×8	2×8	2×8	2×8	6×8	6×8
Chest 3 kg Medicine Ball Throw ^1,2^	2×8	2×8	2×8	2×8		
Overhead 1kg Medicine Ball Throw ^1,2^	2×8	2×8	2×8	2×8	6×8	6×8
Overhead 3kg Medicine Ball Throw ^1,2^	2×8	2×8	2×8	2×8		
CMJ onto a box ^1,2^	1×5	1×5	3×5	3×5	3×5	4×5
Plyometric Jumps above 3 hurdling^1,2^	5×4	5×4	5×4	5×4	2×3	2×3
Sprint Running (m)^1,2^	4×20m	4×20m	3×20m	3×20m	3×20m	3×20m
20m Shuttle Run (MAV)^2^	75%	75%	75%	75%	75%	75%

Exercises	Session 7	Session 8	Session 9	Session 10	Session 11	Session 12

Chest 1 kg Medicine Ball Throw ^1,2^						
Chest 3 kg Medicine Ball Throw ^1,2^	2×5	2×5	3×5	3×5	3×5	2×5
Overhead 1kg Medicine Ball Throw ^1,2^						
Overhead 3kg Medicine Ball Throw ^1,2^	2×8	2×8	3×8	3×8	3×8	
CMJ onto a box ^1,2^	4×5	5×5	5×5	5×5	5×5	4×5
Plyometric Jumps above 3 hurdling^1,2^	3×3	4×3	4×3	4×3	4×3	
Sprint Running (m)^1,2^	4×30m	4×30m	4×30m	4×30m	4×30m	3×40m
20m Shuttle Run (MAV)^2^	75%	TestM	75%	75%	75%	75%

Exercises	Session 13	Session 14	Session 15	Session 16		

Chest 1 kg Medicine Ball Throw ^1,2^						
Chest 3 kg Medicine Ball Throw ^1,2^	2×5	1×5				
Overhead 1kg Medicine Ball Throw ^1,2^		3×8	2×8	2×8		
Overhead 3kg Medicine Ball Throw ^1,2^	3×8					
CMJ onto a box ^1,2^	4×5	2×5	2×4	2×4		
Plyometric Jumps above 3 hurdling^1,2^	4×3	3×3				
Sprint Running (m)^1,2^	3×40m	4×40m	2×30m	2×30m		
20m Shuttle Run (MAV)^2^	75%	75%	75%	75%		

For the Medicine Ball Throwing and Jump onto box the 1^st^ no. corresponds to sets and 2^nd^ corresponds to repetitions. For Sprint Running 1^st^ number corresponds to sets and 2^nd^ corresponds to the distance to run. For 20m Shuttle Run training each girl ran each session (until testM) 75% of maximum individual aerobic volume performed on pre-test and after this testM moment until program end, ran 75% of maximum individual aerobic volume performed on testM. CMJ – Counter movement jump. MAV - maximum individual aerobic volume

1=power strength training protocol (GR).

2=concurrent resistance and endurance training (GCOM).

**Table 2:** Descriptive (mean ± standard deviation) characteristics of the participants during three testing trials (M1, M2 and M3) for all groups

Variable	Group	M1 x±σ	M2 x±σ	M3 x±σ	(M1–M2)	(M2–M3)
Body Weight (kg)	GC	51,5±11,1	53,9±12,7	51,9±12,2	0,39	0,99
	GR	58,9±13,5	59,0±14,1	60,4±15,4	0,95	0,56
	GCOM	54,8±17,1	54,5±18,0	55,2±18,3	0,64	**0,04**

Total Standing Height (cm)	GC	156,8±6,5	158,3±6,9	158,9±6,9	0,06	0,34
	GR	159,4±6,1	159,4±6,0	160,2±6,3	0,14	**0,03**
	GCOM	157,9±8,2	158,0±7,8	158,2±7,9	0,79	0,07

BMI (kg.m^−2^)	GC	20,9±4,0	21,6±4,7	20,9±4,9	0,68	0,65
	GR	23,0±4,1	23,0±4,5	23,2±5,4	0,35	0,62
	GCOM	21,6±5,3	21,6±5,4	21,8±5,6	0,24	0,12

Body Fat (%)	GC	24,34±6,5	24,29±7,8^₮^	22,42±8,8	**0,01**	0,79
	GR	32,14±7,7	30,16±8,2	31,53±8,6^₮^	**0,00**	0,3
	GCOM	26,79±9,9	24,23±10,4^¥^	25,34±11,1	**0,00**	0,3

x – mean; σ - standard deviation; M1 – before training program; M2 – After training program; M3 – After detraining period; p(M1–M2)- p value for comparison between 2^nd^ and 1^st^ moment, p(M2–M3) - p value for comparison between 3^th^ and 2^nd^ moment; GC – Control Group, GR – resistance training group, GCOM - concurrent resistance and endurance training,

₮ - Significant changes between GC and GR;

‡ - Significant changes between GC and GCOM;

¥ - Significant changes between GR and GCOM.

**Table 3:** Mean ± standard deviation of CMVJ, CMSLJ, 1 and 3kg Medicine Ball Throwing, Running Speed and VO_2_ Max at all three testing trials (M1, M2 and M3) for each group

Variable	Group	M1 x±σ	M2 x±σ	M3 x±σ	(M1–M2)	(M2–M3)
1Kg Medicine ball throwing (m)	GC	5,91±0,83	5,76±0,57	5,57±0,52	0,29	0,23
	GR	6,43±1,26	6,80±1,34^₮^	6,73±1,18^₮^	0,08	0,06
	GCOM	6,14±1,00	6,67±1,16^‡^	6,69±1,18^‡^	**0,00**	0,78

3Kg Medicine ball throwing (m)	GC	3,79±0,50	3,76±0,43	3,59±0,50	0,37	**0,03**
	GR	3,93±0,73	4,29±0,74^₮^	4,67±1,34^₮^	**0,01**	0,23
	GCOM	3,89±0,64	4,25±0,73^‡^	4,25±0,74^‡^	**0,00**	0,49

CM Vertical Jump (cm)	GC	0,26±0,07	0,26±0,06	0,20±0,04	0,13	**0,02**
	GR	0,25±0,06	0,27±0,07	0,26±0,06^₮^	**0,01**	**0,02**
	GCOM	0,25±0,06	0,28±0,08	0,24±0,06^‡^	**0,00**	**0,00**

CM Standing Long Jump (m)	GC	1,32±0,23	1,29±0,20	1,31±0,31	0,17	0,50
	GR	1,31±0,24	1, 32±0,26	1,27±0,29	**0,04**	0,23
	GCOM	1,30±0,26	1,37±0,22	1,31±0,30	**0,01**	**0,05**

Running Speed 20m (s)	GC	4,42±0,44	4,20±0,36	4,25±0,36	0,10	**0,03**
	GR	4,91±0,57	4,28±0,38	4,32±0,40	**0,00**	0,86
	GCOM	4,80±0,53	4,25±0,34	4,33±0,39	**0,00**	**0,00**

VO_2_Max (mL.kg^−1^.min^−1^)	GC	40,8±4,05	41,0±4,27	45,0±8,20	**0,05**	0,21
	GR	39,2±4,29	40,7±3,98	42,0±6,84	0,13	0,58
	GCOM	42,5±4,37	43,7±4,09^‡¥^	41,9±5,80	**0,01**	0,43

x – mean; σ- standard deviation; CM – counter movement; M1 – before training program; M2 – After training program; M3 – After detraining period; p (M1–M2) - p value for comparison between 2^nd^ and 1^st^ moment, p (M2–M3) - p value for comparison between 3^th^ and 2^nd^ moment; GC – Control Group, GR – resistance training group, GCOM - concurrent resistance and endurance training,

₮ - Significant changes between GC and GR;

‡ - Significant changes between GC and GCOM;

¥ - Significant changes between GR and GCOM.

## References

[b1-jhk-29a-93] Abernethy P, Quigley B (1993). Concurrent Strength and endurance training of the elbow extensors. J Strength Cond Res.

[b2-jhk-29a-93] Anderson LB, Haraldsdottir J (1995). Coronary heart disease risk factors, physical activity, and fitness in young Danes. Med Sci Sports Exerc.

[b3-jhk-29a-93] Christodoulos AD, Flouris AD, Tokmakidis SP (2006). Obesity and physical fitness of pre-adolescent children during the academic year and the summer period: effects of organized physical activity. J Child Health Care.

[b4-jhk-29a-93] Duke PM, Litt IR, Gross RT (1980). Adolescents' self-assessment of sexual maturation. Pediatrics.

[b5-jhk-29a-93] Faigenbaum AD, Mediate P (2006). Effects of Medicine Ball Training on Fitness Performance of High-School Physical Education Students. Phys.

[b6-jhk-29a-93] Faigenbaum AD (2007). State of the Art Reviews: Resistance Training for Children and Adolescents: Are There Health Outcomes?. Am J Lifestyle Med.

[b7-jhk-29a-93] Faigenbaum AD, Milliken LA, Loud LR, Burak BT, Doherty CL, Westcott WL (2002). Comparison of 1 and 2 Days Per Week of Strength Training in Children. Res Q Exerc Sport.

[b8-jhk-29a-93] Faigenbaum AD, Kraemer WJ, Blimkie CJ, Jeffreys I, Micheli LJ, Nitka M, Rowland TW (2009). Youth resistance training: updated position statement paper from the national strength and conditioning association. J Strength Cond Res.

[b9-jhk-29a-93] Faigenbaum AD, Westcott WL, Micheli LJ, Outerbridge AR, Long CJ, LaRosa-Loud R, Zaichkowskt LD (1996). The Effects of Strength Training and Detraining on Children. J Strength Cond Res.

[b10-jhk-29a-93] Faigenbaum AD, Westcott WL, Loud RL, Long CJ (1999). The Effects of Different Resistance Training Protocols on Muscular Strength and Endurance Development in Children. J Pediatr.

[b11-jhk-29a-93] Garrido N, Marinho DA, Reis VM, van den Tillaar R, Costa AM, Silva AJ, Marques MC (2010). Does concurrent dry land strength and aerobic training inhibit strength and swimming performance in young competitive swimmers?. J Sports Sci Med.

[b12-jhk-29a-93] Hunter G, Demment R, Miller D (1987). Development of strength and maximum oxygen uptake during simultaneous training for strength and endurance. Sports Med Phys Fitness.

[b13-jhk-29a-93] Izquierdo M, Exposito RJ, Garcia-Pallare J, Medina L, Villareal E (2010). Concurrent Endurance and Strength Training Not to Failure Optimizes Performance Gains. Sci Sports Exerc.

[b14-jhk-29a-93] Kraemer WJ, Patton JF, Gordon SE (1995). Compatibility of high-intensity strength and endurance training on hormonal and skeletal muscle adaptations. J Appl Physiol.

[b15-jhk-29a-93] Léger LA, Mercier D, Gadoury C, Lambert J (1988). The multistage 20 meter shuttle run test for aerobic fitness. J Sports Sci.

[b16-jhk-29a-93] Linthorne NP (2001). Analysis of standing vertical jumps using a force platform. Am J Physics.

[b17-jhk-29a-93] Lubans DR, Sheaman C, Callister R (2010). Exercise adherence and intervention effects of two school-based resistance training programs for adolescents. Prev Med.

[b18-jhk-29a-93] Malina R (2001). Physical Activity and Fitness: Pathways from Childhood to Adulthood. Am J Hum Biol.

[b19-jhk-29a-93] Marfell-Jones M, Olds T, Stewart A, Carter L (2006). International standards for anthropometric assessment.

[b20-jhk-29a-93] Mujika I, Padilla S (2001). Cardiorespiratory and metabolic characteristics of detraining in humans. Med Sci Sports Exerc.

[b21-jhk-29a-93] Nielsen GA, Andersen LB (2003). The association between high blood pressure, physical fitness, and body mass index in adolescents. Prev Med.

[b22-jhk-29a-93] Ozmun JC, Mikesky AE, Surburg P (1994). Neuromuscular adaptations following prepubescent strength training. Med Sci Sports Exerc.

[b23-jhk-29a-93] Sale DG, McDougall JD, Jacobs I, Garner S (1990). Interaction between concurrent strength and endurance training. J Appl Physiol.

[b24-jhk-29a-93] Sharma M (2006). School-based interventions for Childhood and Adolescent Obesity. Obes Res.

[b25-jhk-29a-93] Silva EF, Oliveira MA, Mendes EL (2010). Influence of vacation time in physical fitness for health in scholars. J Health Sci Ins.

[b26-jhk-29a-93] Simons-Morton BG, Taylor WC, Snider SA (1994). Observed levels of elementary and middle school children's physical activity during physical education classes. Prev Med.

[b27-jhk-29a-93] Simons-Morton BG, Taylor WC, Snider SA (1993). The physical activity of fifthgrade students during physical education. Am J Public Health.

[b28-jhk-29a-93] Strong WB, Malina RM, Blimkie CJ (2005). Evidence based physical activity for school-age youth. J Pediatr.

[b29-jhk-29a-93] Sweeting HN (2007). Measurement and definitions of obesity in childhood and adolescence: a field guide for the uninitiated. Nutr J.

[b30-jhk-29a-93] Tsolakis CK, Vagenas GK, Dessypris AG (2004). Strength adaptations and hormonal responses to resistance training and detraining in preadolescent males. J Strength Cond Res.

[b31-jhk-29a-93] Twisk JW, Kemper HC, van Mechelen W (2000). Tracking of activity and fitness and the relationship with cardiovascular disease risk factors. Med Sci Sports Exerc.

[b32-jhk-29a-93] Van den Tillaar R, Marques MC (2009). Effect of two different throwing training programs with same workload on throwing performance with soccer ball. Int J Sports Phys Perf.

[b33-jhk-29a-93] Volpe S, Walberg-Rankin J, Rodman K, Sebolt D (1993). The Effect of Endurance Running on Training Adaptations in woman Participating in a Weight Lifting Program. Restriction on Strength Performance. Nat Strength Cond Assoc J.

[b34-jhk-29a-93] Watts K, Beye P, Siafarikas A (2004). Exercise training normalises vascular dysfunction and improves central adiposity in obese adolescents. J Am Coll Cardiol.

